# Pre-sleep protein supplementation in professional cyclists during a training camp: a three-arm randomized controlled trial

**DOI:** 10.1080/15502783.2023.2166366

**Published:** 2023-01-15

**Authors:** Pedro L. Valenzuela, Lidia B. Alejo, Almudena Montalvo-Pérez, Diego Ojanguren, Miguel Górriz, Itziar Pagola, Laureano M. Ozcoidi, Alejandro Lucia, David Barranco-Gil

**Affiliations:** a Physical Activity and Health Research Group (PaHerg), Research Institute of Hospital 12 de Octubre (imas12), Madrid, Spain; b Department of Systems Biology, University of Alcala, Madrid, Spain; c Faculty of Sport Sciences, Universidad Europea de Madrid, Madrid, Spain; d Caja Rural Professional Team, Navarra, Spain

**Keywords:** Recovery, performance, nutrition, cycling, supplement

## Abstract

**Background:**

The effects of pre-sleep protein supplementation on endurance athletes remain unclear, particularly whether its potential benefits are due to the timing of protein intake or solely to an increased total protein intake. We assessed the effects of pre-sleep protein supplementation in professional cyclists during a training camp accounting for the influence of protein timing.

**Methods:**

Twenty-four professional U23 cyclists (19 ± 1 years, peak oxygen uptake: 79.8 ± 4.9 ml/kg/min) participated in a six-day training camp. Participants were randomized to consume a protein supplement (40 g of casein) before sleep (n = 8) or in the afternoon (n = 8), or an isoenergetic placebo (40 g of carbohydrates) before sleep (n = 8). Indicators of fatigue/recovery (Hooper index, Recovery–Stress Questionnaire for Athletes, countermovement jump), body composition, and performance (1-, 5-, and 20-minute time trials, as well as the estimated critical power) were assessed as study outcomes.

**Results:**

The training camp resulted in a significant (p < 0.001) increase in training loads (*e.g*. training stress score of 659 ± 122 per week during the preceding month versus 1207 ± 122 during the training camp), which induced an increase in fatigue indicators (e.g. time effect for Hooper index p < 0.001) and a decrease in performance (e.g. time effect for critical power p = 0.002). Protein intake was very high in all the participants (>2.5 g/kg on average), with significantly higher levels found in the two protein supplement groups compared to the placebo group (p < 0.001). No significant between-group differences were found for any of the analyzed outcomes (all p > 0.05).

**Conclusions:**

Protein supplementation, whether administered before sleep or earlier in the day, exerts no beneficial effects during a short-term strenuous training period in professional cyclists, who naturally consume a high-protein diet.

## 1. Introduction

Strong evidence supports the effectiveness of protein supplementation to increase muscle mass, strength, and performance in healthy subjects, particularly when combined with exercise training [1–3]. Although extensive research has studied the effects of protein supplementation in resistance-trained individuals [1,4], endurance athletes might also benefit from this nutritional intervention, especially during periods of high energy demands (*e.g*. strenuous training) [5,6]. Notably, protein supplementation might attenuate muscle damage and facilitate skeletal muscle mass remodeling in endurance athletes [7,8], which could potentially improve tolerance to training loads and subsequent physiological adaptations in these individuals [8–10]. However, some authors have reported no beneficial effect of protein supplementation on endurance training-induced adaptations [11,12] or on performance and recovery indicators in periods of strenuous endurance training (e.g. during a training camp) in professional cyclists [13].

Pre-sleep protein supplementation––particularly casein, characterized by a slower absorption and digestion than other protein sources such as whey––is gaining attention among athletes [14]. Protein ingested prior to sleep is effectively digested and absorbed during overnight, thereby increasing muscle protein synthesis and improving net protein balance [15]. A recent systematic review indeed suggested that this strategy might help to promote exercise training-induced adaptations (*e.g*. increases in muscle mass and strength) [16]. There is, however, controversy, as some studies have found no beneficial effects of pre-sleep protein supplementation [17–20]. Moreover, scarce evidence exists on the potential benefits of pre-sleep protein supplementation in endurance athletes undergoing periods of strenuous training (*e.g*. training camps or multistage cycling races) [18]. It is also worth noting that pre-sleep protein supplementation has been reported to enhance whole-body protein balance when exercise training is performed late in the day [15,21], but whether it could also be beneficial if exercise is performed in the morning remains to be elucidated. Moreover, most research to date has analyzed the benefits of pre-sleep protein supplementation compared to a non-protein placebo instead of a protein supplement provided at another time of the day. It therefore remains unclear whether the benefits of pre-sleep protein supplementation are solely due to an increased total protein intake or if changes in the timing of intake might also play a role [14,22].

The aim of this study was to analyze the effects of pre-sleep protein supplementation––compared to both the same supplement provided at different timing and an isoenergetic placebo––on recovery/fatigue indicators, body composition, and field-based performance in professional cyclists during a training camp.

## 2. Material and methods

### Participants

Twenty-four U23 cyclists (age: 19 ± 1 years, peak oxygen uptake [VO_2peak_]: 79.8 ± 4.9 ml/kg/min, peak power output [PPO]: 6.8 ± 0.4 W/kg) from the same team volunteered to participate in the study. They all competed at the national or international level and could be considered of the highest performance level (*i.e*. level 5) attending to the guidelines proposed by de Pauw et al. [23]. To be enrolled in the study, cyclists had to be free of musculoskeletal injuries or other conditions that could hinder their participation. None of the cyclists were taking any type of medication or supplement (other than the relevant supplement as per group assignment) and they were instructed to maintain their normal diet during the study period. They were informed of the study procedures and provided written informed consent. The study was approved by the Ethical Committee of Alcorcón University Hospital (approval number 20/201), and all procedures were conducted following the standards established by the Declaration of Helsinki and its later amendments.

### Experimental design and nutritional intervention

**Figure 1.**
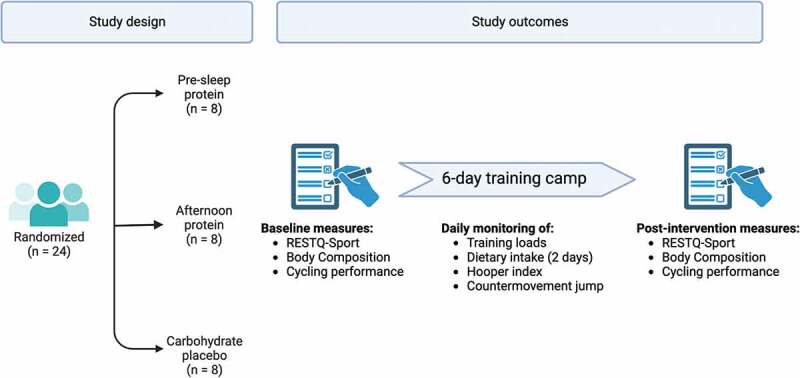
Schematic summary of the study design. Abbreviations: RESTQ-Sport, Recovery-Stress Questionnaire for Athletes.

The study took place during a strenuous six-day training camp at the end of the “pre-season” period, more than two months after the last competition of the previous season. The protocol followed a double-blinded, parallel-group, three-arm, randomized controlled design. The study design is summarized in Figure 1.

Participants were randomized by an external researcher into one of the three study groups (N = 8 each) using a specific software (https://www.sealedenvelope.com) consuming: 40 g of casein (Victory Endurance, Weider, Madrid, Spain) at night (~10.30 pm, corresponding to *~*2 hours after dinner and ~30 minutes before sleep) (*Pre-sleep PRO*); or 40 g of casein (Victory Endurance, Weider, Madrid, Spain) in the afternoon (~6.30 pm, at least ~2 hours after the post-training meal and ~2 hours before dinner) (*Afternoon PRO*); or 40 g of an isoenergetic placebo (maltodextrin [Life Pro endurance maltodextrin, Index Sport Nutrition, Madrid, Spain]) ~30 minutes before sleep (*Placebo*).

The three supplements were dispensed in identical opaque bottles diluted in ~500 ml of water and were provided along with cocoa flavor so that they were similar in appearance and taste. All cyclists trained during morning hours (~09.00 am to ~13.30 pm) and consumed lunch after training. Thus, more than four hours had elapsed between the end of training and supplement intake in all the participants, including those in the *Afternoon PRO* group. Participants and researchers involved in supplement administration or outcome assessment were blinded to group assignment throughout the entire duration of the study.

### Measurements

#### Laboratory performance indicators

Participants’ VO_2peak_ and PPO were determined as descriptive (non-outcome) variables one week before the training camp as explained elsewhere [24]. Participants performed a maximal incremental cycling test on their own bikes, which were placed on a validated indoor trainer (Hammer, CycleOps, Madison, WI) [25]. The initial workload was 75 W and increased by 5 W every 12 seconds until volitional exhaustion or when pedaling cadence could not be maintained above 60 rpm. Gas exchange data were collected breath-by-breath (Ultima Series Medgraphics, Cardiorespiratory Diagnostics, Saint Paul, MN). PPO was defined as the highest power output value reached during the test, and VO_2peak_ was defined as the highest VO_2_ (mean of 10 s) attained during the test.

#### Training load control

Daily training loads were monitored during the month preceding the study as well as during the training camp. Power output data were registered during all training sessions with a power meter (Shimano Dura-Ace FCRC9100-P, Shimano, Sakia, Japan). Training volume (total time/distance covered), work (kJ spent), and the training stress score (TSS) were also recorded for each session. The TSS was computed as:

$$TSS=\left(\left(t^{*}NP^{*}IF\right)/\left(FTP^{*}3600\right)\right){\;}^{*}100$$

where t = duration (s), NP = normalized power output, and IF = intensity factor calculated as NP/functional threshold power (FTP). NP was computed as the 30-second moving average of the power output attained during the relevant training session, and is thought to represent the power output an individual could sustain if intensity was maintained constant without any variability. FTP was calculated as 95% of the highest power output attained for a 20-minute effort during the previous four weeks [26]. Data from all the sessions were analyzed using a software WKO (WKO5 Build 576; TrainingPeaks LLC, Boulder, CO)

#### Nutritional assessment

Participants completed a two-day––one weekday and one weekend day––food report during the training camp and captured photos of all the food ingested during these two days. Nutritional intake was analyzed using a software tool (DietoPro, Valencia, Spain), and the mean intake of macronutrients over two days was computed and averaged.

#### Recovery and fatigue

The Hooper’s index––a practical and valid indicator of athletes’ internal load [27]––was recorded every morning post waking up [27]. Participants rated their perceived sleep quality, stress, fatigue, and delayed-onset muscle soreness (DOMS) on a 7-point scale––with minimum (=1) and maximum scores (=7) representing “very, very good” and “very, very poor,” respectively; a total score was computed by summing the four components.

Every morning before training, participants performed three countermovement jumps (CMJ), and the mean height was calculated using a contact platform (Chronojump, Boscosystem, Barcelona, Spain) [28]. They were instructed to perform a downward movement to reach approximately 90° of knee flexion while maintaining their hands on the hips. Cyclists were also instructed not to flex their knees during the flight in order to avoid overestimation of the flight time. Changes in jumping ability have been previously reported to be highly correlated to metabolic––increases in lactate and ammonia––and mechanic measures of fatigue [29].

The stress/recovery balance of the cyclists was assessed using the validated Spanish version [30] of the Recovery-Stress Questionnaire for Athletes (RESTQ-Sport) the day before and after the training camp. The RESTQ-Sport is responsive to acute and chronic changes in training loads, representing a valid tool for the identification of overarching and overtraining states [31]. It is organized around two major theoretical constructs, stress and recovery, and contains 77 items pertaining to 19 subscales [32]. Each subscale contains four items that are measured using a Likert-type scale with anchors ranging from 0 (“never”) to 6 (“always”).

#### Body composition

Body mass and composition were assessed the day before and after the training camp using bioelectrical impedance analysis (InBody 720, Inbody USA, Cerritos, CA) [33] after at least 24 hours of rest, and participants were instructed to attend to the laboratory euhydrated and to empty their urinary bladder at least 30 min before assessment. Height was also measured, using a stadiometer (Harpender, Holtain Limited, Crymych, UK).

#### Field-based performance

The day before and after the training camp (following body composition assessment), participants performed three separate field-based time trials of 1, 5, and 20 minutes––consistently in this order––and the mean power output attained during each trial was registered. The trials were interspersed by 30 minutes of passive recovery, as done in previous studies [34–37]. No specific pacing strategy was recommended, although participants were encouraged to achieve the highest mean power output possible during each trial. Critical power (CP) was calculated as explained elsewhere using the “1/t method” [38]. All trials were performed on the same uphill climb (distance = 16.3 km, average inclination = 5.9%).

### Statistical analysis

Data are shown as mean ± standard deviation. The normal distribution (Shapiro – Wilk test) and homoscedasticity (Levene’s test) of the data were checked before any statistical treatment. Between-group differences in variables assessed at a single time point were determined with a one-way ANOVA, whereas differences in those assessed at several time points were determined with a mixed-model ANOVA, setting group and time as the between- and within-subject factor, respectively. In order to reduce the risk of type I error, post hoc differences (Bonferroni test) were only checked when a significant group-by-time interaction effect was found. Effect sizes (partial eta squared, ηp^2^) were computed for the study outcomes. Analyses were performed using SPSS (version 23.0, IBM, NY) and the significance level was set at 0.05.

## 3. Results

All participants completed all procedures and training sessions and were included in the analyses. No adverse effect was reported. Compared with the preceding month, the training camp induced a significant increase in the training loads of the participants, as reflected by raises in weekly training time (14.9 ± 2.7 versus 23.9 ± 0.8 hours, respectively; p < 0.001), load (TSS: 659 ± 122 versus 1207 ± 122 TSS, respectively; p < 0.001), distance (423 ± 81 versus 684 ± 25 km, respectively; p < 0.001), or work (9765 ± 2154 versus 15,545 ± 1975 kJ, respectively; p < 0.001).

No between-group differences were found for baseline variables, including age (19 ± 1, 20 ± 2 and 19 ± 2 years for *pre-sleep PRO, Afternoon PRO,* and *placebo*, respectively; p = 0.556), body mass (65.0 ± 6.3, 68.9 ± 5.8, and 63.5 ± 4.9 kg, respectively; p = 0.178), body mass index (20.9 ± 1.3, 20.9 ± 1.1, and 20.1 ± 1.4 kg/m^2^, p = 0.344), VO_2peak_ (79.4 ± 4.8, 80.0 ± 3.7, and 80.0 ± 6.5 ml/kg/min, p = 0.961), or PPO (6.8 ± 0.4, 6.8 ± 0.4, and 6.8 ± 0.5 W/kg, respectively; p = 0.975).

Participants’ macronutrient intake during the training camp is shown in Table 1. The three groups showed similar values of total energy, carbohydrate, and fat intake, respectively. However, protein intake was higher (p < 0.001) in the *pre-sleep PRO* and *Afternoon PRO* groups compared with *Placebo*.

**Table 1.** Total energy and macronutrient intake by group.

Outcome	*Pre-sleep PRO*	*Afternoon PRO*	*Placebo*	P-value for group effect	Effect size (ηp^2^)
Total energy (Kcal/d)	3381 ± 409	3291 ± 520	3114 ± 273	0.436	0.076
Carbohydrates (g/d)	498 ± 212	458 ± 166	411 ± 74	0.564	0.053
Carbohydrates (g/kg/day)	7.74 ± 3.51	6.75 ± 2.82	6.49 ± 1.20	0.625	0.044
Carbohydrates (%)	59.8 ± 6.7	60.9 ± 5.4	62.1 ± 5.7	0.732	0.029
Protein (g/d)	231 ± 20*	223 ± 12*	164 ± 13	<0.001	0.816
Protein (g/kg/d)	3.57 ± 0.35*	3.26 ± 0.35*	2.59 ± 0.30	<0.001	0.626
Protein (%)	29.1 ± 4.9	29.7 ± 5.9	24.7 ± 1.8	0.079	0.214
Fat (g/d)	88.0 ± 37.2	89.9 ± 20.3	82.3 ± 21.1	0.846	0.016
Fat (g/kg/d)	1.35 ± 0.52	1.32 ± 0.34	1.31 ± 0.38	0.986	0.001
Fat (%)	10.8 ± 4.1	12.5 ± 2.3	12.8 ± 4.3	0.502	0.063

Data are shown as mean ± SD and expressed as units per day. Abbreviations: *Afternoon PRO*, protein supplementation in the afternoon; *Pre-sleep PRO*, protein supplementation before sleep. Symbol: * Significant post-hoc differences (p < 0.001) compared to Placebo.

### Recovery and fatigue

**Figure 2.**
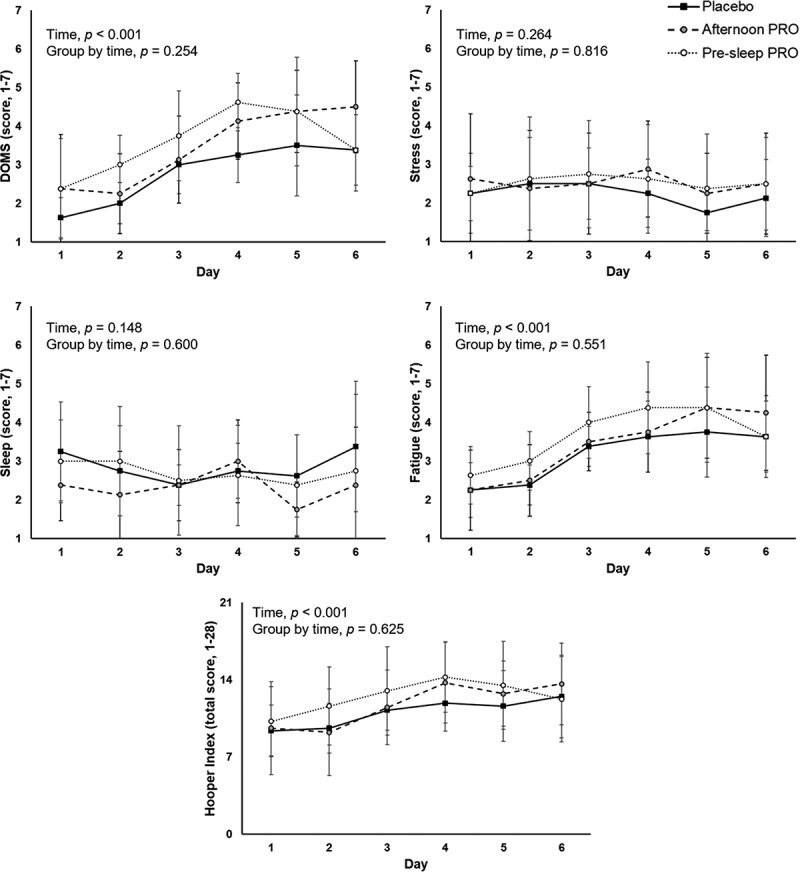
Differences between study groups on the Hooper index sub-items and total score reported daily during the training camp. Abbreviations: *Afternoon PRO*: protein supplementation provided in the afternoon; DOMS, delayed-onset muscle soreness; *Pre-sleep PRO*, protein supplementation before sleep.

**Figure 3.**
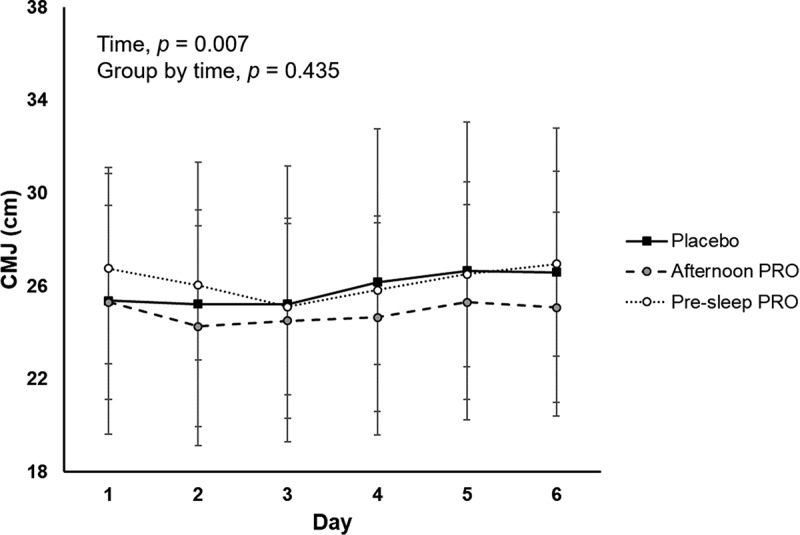
Differences between study groups on the height attained during a countermovement jump (CMJ). Abbreviations: *Afternoon PRO*, protein supplementation provided during the afternoon; *Pre-sleep PRO*, protein supplementation before sleep.

A significant time effect was observed for the total Hooper index score (p < 0.001) as well as for specific items of fatigue (p < 0.001) and DOMS (p < 0.001), all of which increased over time––except for sleep (p = 0.148) or stress (p = 0.264); yet, no significant group-by-time interaction effect was observed for any of the items (Figure 2). No significant group effect was found for the average Hooper index reported during the training camp (Table 2). On the other hand, a significant time effect was found for CMJ (p = 0.007), yet with no significant group-by-time interaction (p = 0.435, ηp^2^ = 0.086) (Figure 3). No significant group-by-time effect was found for RESTQ scores (Table 3).

**Table 2.** Hooper index (average scores reported during the training camp) by group.

Hooper index scores	*Pre-sleep PRO*	*Afternoon PRO*	*Placebo*	P-value for group effect	Effect size (ηp^2^)
**Individual items (0 to 7)**					
Stress	2.5 ± 1.1	2.5 ± 1.2	2.2 ± 0.7	0.803	0.046
Sleep	2.7 ± 1.2	2.3 ± 0.8	2.9 ± 0.5	0.475	0.073
DOMS	3.6 ± 0.6	3.5 ± 0.7	2.8 ± 0.6	0.043*	0.385
Fatigue	3.7 ± 0.7	3.4 ± 0.8	3.2 ± 0.5	0.364	0.070
**Total (0 to 28)**	12.5 ± 2.9	11.8 ± 2.9	11.0 ± 2.0	0.564	0.071

Data are shown as mean ± SD. Although a significant group effect (p = 0.043) was observed for delayed-onset muscle soreness (DOMS), post hoc analyses revealed no specific between-group differences. Abbreviations: *Afternoon PRO*, protein supplementation in the afternoon; DOMS, delayed-onset muscle soreness; *Pre-sleep PRO*, protein supplementation before sleep. Symbol: * no between-group differences in post-hoc analyses

**Table 3.** Results of the Recovery–Stress Questionnaire for Athletes (RESTQ-Sport) by group.

Item	*Pre-sleep PRO*		*Afternoon PRO*		*Placebo*		P-value for time effect	P-value for group-by-time effect	Effect size (ηp^2^)
	Baseline	Post	Baseline	Post	Baseline	Post			
General_Stress	0.43 ± 0.69	0.34 ± 0.37	0.68 ± 0.57	1.25 ± 0.51	0.78 ± 0.57	0.71 ± 0.61	0.734	0.153	0.164
Emotional stress	1.03 ± 0.91	0.81 ± 0.81	1.09 ± 0.87	1.09 ± 0.87	1.21 ± 0.79	1.00 ± 0.81	0.535	0.906	0.009
Social stress	0.78 ± 0.93	0.65 ± 0.59	0.81 ± 0.76	0.65 ± 0.26	0.65 ± 0.56	0.50 ± 0.61	0.337	0.995	0.000
Conflicts pressure	2.03 ± 1.28	1.71 ± 1.19	2.12 ± 1.52	2.40 ± 0.54	1.87 ± 0.89	1.62 ± 0.75	0.686	0.517	0.061
Fatigue	1.28 ± 1.01	1.62 ± 0.66	1.18 ± 0.63	1.90 ± 0.80	1.53 ± 1.44	1.46 ± 0.86	0.085	0.247	0.125
Lack of energy	0.84 ± 0.85	1.00 ± 0.95	1.34 ± 1.01	1.15 ± 0.68	1.06 ± 0.82	1.18 ± 0.86	0.874	0.730	0.030
Physical complaints	0.78 ± 0.55	1.06 ± 0.54	0.75 ± 0.71	1.15 ± 0.70	1.12 ± 0.86	1.37 ± 1.03	0.057	0.910	0.009
Success	3.40 ± 1.00	2.40 ± 1.09	2.87 ± 1.15	2.31 ± 1.23	3.03 ± 0.82	2.81 ± 0.90	0.004**	0.243	0.126
Social recovery	3.25 ± 1.50	3.71 ± 0.60	2.96 ± 1.63	3.50 ± 1.25	3.00 ± 1.06	3.93 ± 1.09	0.034*	0.769	0.025
Physical recovery	2.93 ± 0.81	3.12 ± 1.12	3.00 ± 1.63	2.96 ± 1.27	3.28 ± 1.41	3.06 ± 1.03	0.932	0.794	0.022
General well being	3.65 ± 1.20	4.68 ± 1.03	3.75 ± 1.37	4.43 ± 0.99	4.21 ± 0.80	4.68 ± 1.72	0.016*	0.852	0.015
Sleep quaity	3.56 ± 0.88	3.59 ± 0.58	4.03 ± 0.93	3.68 ± 1.46	4.15 ± 0.37	3.46 ± 1.12	0.165	0.461	0.071
Disturbed breaks	0.84 ± 0.46	1.03 ± 0.77	0.59 ± 0.58	1.37 ± 2.32	0.84 ± 0.90	1.28 ± 1.85	0.159	0.753	0.027
Emotional exhaustion	0.68 ± 0.47	0.46 ± 0.66	0.84 ± 0.54	1.59 ± 2.12	0.81 ± 0.71	1.43 ± 1.19	0.229	0.401	0.083
Injury	1.28 ± 0.89	1.93 ± 0.88	1.87 ± 1.73	2.59 ± 1.42	1.65 ± 0.85	2.12 ± 0.98	0.007**	0.877	0.012
Being in shape	3.71 ± 1.04	3.59 ± 1.43	4.25 ± 1.19	3.90 ± 1.58	3.59 ± 1.36	3.84 ± 1.49	0.769	0.613	0.046
Personal accomplishment	3.12 ± 1.06	2.96 ± 1.31	2.93 ± 0.94	2.90 ± 1.28	3.15 ± 0.51	3.43 ± 0.41	0.887	0.701	0.033
Self efficacy	3.65 ± 1.48	3.87 ± 1.70	3.90 ± 1.31	3.90 ± 1.41	3.34 ± 1.15	4.03 ± 1.44	0.232	0.517	0.061
Sefl regulation	3.62 ± 1.24	3.68 ± 1.71	3.96 ± 1.29	3.90 ± 1.26	4.12 ± 1.14	4.25 ± 0.55	0.858	0.944	0.005

Data are shown as mean ± SD. Abbreviations: *Afternoon PRO*, protein supplementation in the afternoon; *Pre-sleep PRO*, protein supplementation before sleep. Symbols for significant time effect: *p < 0.05, **p < 0.01.

### Body composition

No significant time (p = 0.445) or group-by-time interaction effect (p = 0.057) was found for fat mass (Table 4). A significant time effect (p < 0.001, p = 0.001, and p = 0.001, respectively) and a group-by-time interaction effect (p = 0.043, p = 0.018, and p = 0.008, respectively) were found for body mass, fat-free mass, and muscle mass. However, post-hoc analyses revealed no significant differences between groups at any specific time point.

**Table 4.** Body composition by group.

Variable	*Pre-sleep PRO*		*Afternoon PRO*		Placebo		P-value for time effect	P-value for group-by-time effect	Effect size (ηp^2^)
	Baseline	Post	Baseline	Post	Baseline	Post			
Body mass (kg)	65.0 ± 6.3	65.3 ± 6.4	68.9 ± 5.8	70.2 ± 6.2	63.5 ± 4.9	64.3 ± 5.0	<0.001***	0.043^#^	0.259
Fat mass (kg)	6.0 ± 1.0	6.2 ± 1.2	7.6 ± 1.5	7.2 ± 1.6	6.4 ± 1.1	6.4 ± 1.1	0.445	0.057	0.238
Fat-free mass (kg)	59.0 ± 6.4	59.1 ± 7.0	61.3 ± 5.2	63.0 ± 5.7	57.2 ± 4.4	57.9 ± 4.7	0.001**	0.018^#^	0.318
Muscle mass (kg)	33.6 ± 4.0	33.6 ± 4.2	34.8 ± 3.1	35.8 ± 3.4	32.3 ± 2.7	32.7 ± 2.8	0.001**	0.008^##^	0.367

Data are shown as mean ± SD. Abbreviations: *Afternoon PRO*, protein supplementation in the afternoon; *Pre-sleep PRO*, protein supplementation before sleep. Symbols for significant time effect: **p < 0.01, ***p < 0.001. Symbols for a significant group-by-time effect: ^#^p < 0.05, ^##^p < 0.01. There were no between-group differences in post-hoc analyses.

### Performance

A significant time effect was observed for the mean power output in the 5-minute (p < 0.002) as well as for the estimated CP (p = 0.004), which tended to decrease after the training camp (Table 5). Yet, no significant group-by-time interaction was found for any performance indicator.

**Table 5.** Field-based cycling performance by group.

Outcome	*Pre-sleep PRO*		*Afternoon PRO*		Placebo		P-value for time effect	P-value for group-by-time effect	
	Baseline	Post	Baseline	Post	Baseline	Post			Effect size (ηp^2^)
1-min time trial (W)	646 ± 78	631 ± 74	665 ± 71	681 ± 79	624 ± 56	614 ± 74	0.729	0.233	0.129
5-min time trial (W)	418 ± 31	399 ± 17	445 ± 42	428 ± 50	411 ± 24	395 ± 41	0.002**	0.966	0.003
20-min time trial (W)	349 ± 27	344 ± 21	370 ± 31	344 ± 21	350 ± 22	345 ± 34	0.119	0.999	0.000
Critical power (W)	344 ± 24	334 ± 16	369 ± 33	355 ± 39	344 ± 20	335 ± 33	0.004**	0.858	0.015

Data are shown as mean ± SD. Abbreviations: *Afternoon PRO*, protein supplementation in the afternoon; *Pre-sleep PRO*, protein supplementation before sleep. Symbols for significant time effect: **p < 0.01.

## 4. Discussion

The main finding of the present study was that protein supplementation––whether administered before sleep or earlier in the day––does not seem to induce beneficial effects on markers of recovery, body composition, or performance compared to an isoenergetic carbohydrate placebo during a short-term period of strenuous training loads in professional cyclists who naturally consume a high-protein diet (>2.5 g/kg/day on average in our participants), as previously documented and corroborated here [39–43].

The acute increase in training loads usually imposed by strenuous situations such as training camps or multistage races represents a physiological challenge for endurance athletes. In this effect, training loads increased approximately two-fold during the six-day training camp compared with the preceding month, which resulted in an increased perceived fatigue, muscle soreness, and a reduced performance in all the study participants. In this effect, protein supplementation might potentially help to accelerate muscle remodeling and recovery during strenuous training [44]. Moreover, the high energy demands of endurance athletes can lead to the oxidation of muscle protein as a fuel (*i.e*. muscle catabolism), and indeed endurance athletes show higher protein requirements than the general population [45]. Protein supplementation might therefore be important in the promotion of recovery as well as in training-induced adaptations in endurance athletes [6]. However, the present study failed to find such benefits.

Some evidence supports a beneficial effect of protein supplementation in situations of strenuous endurance training. Huang et al. [7] observed lower levels of muscle damage markers (*e.g*. serum creatine kinase levels) along with improved endurance performance in marathon runners who ingested a protein supplement compared to carbohydrate supplementation. Other studies have reported that replacing part of a carbohydrate supplement with a protein content improves markers of recovery and enhances nitrogen balance after exhaustive exercise in cyclists [46–48]. Similarly, protein supplementation has been reported to improve performance and reduce markers of muscle damage during a strenuous one-week training camp in runners compared to an energy-matched carbohydrate supplement [8], as well as to promote adaptations (*e.g*. increased lean mass, muscle oxidative capacity, and performance) in response to long-term endurance training [9,49,50]. It must be noted, however, that other authors found no beneficial effect of protein supplementation on performance or recovery indicators during a six-day training camp in professional cyclists [13]. In the same line, Jonvik et al. [11] and Robertson et al. [12] reported that protein supplementation did not promote adaptations to endurance training in active males compared to a placebo.

Another major finding of the present study was the lack of beneficial effects of pre-sleep protein supplementation when compared not only with the same protein supplement provided earlier in the day but also with an isoenergetic non-protein placebo. Pre-sleep protein supplementation has received attention in recent years due to its potential to enhance recovery and promote training-induced adaptations [14–16,51]. However, in line with our results, findings from previous studies have also failed to find a beneficial effect from this strategy. For instance, Antonio et al. observed no beneficial effects of pre-sleep protein supplementation during 8 weeks on training-induced adaptations compared to the same quantity of casein administered in the morning [52]. Other studies have also found no beneficial effects of pre-sleep protein compared to a non-protein control. A study in older adults reported that protein supplementation both after exercise and before sleep induced no benefits on resistance training-induced gains compared to carbohydrate ingestion [17]. Pre-sleep protein supplementation has also been reported not to enhance recovery compared to carbohydrate ingestion after an evening bout of resistance exercise [20] or a muscle-damaging exercise protocol (100 drop jumps) performed in the morning [19], or during a one-week training camp in which a group of runners trained both during the morning and afternoon [18]. Thus, further research is needed to confirm the eventual benefits of pre-sleep protein supplementation compared to a placebo and particularly compared with protein supplementation earlier in the day. Moreover, although it could be argued that the benefits of pre-sleep protein supplementation could be greater if exercise is performed in the evening [14,15], evidence is also needed to confirm whether exercise timing plays an influence on the effects of protein supplementation, as there is no consistent research supporting meaningful differences when protein is ingested immediately after exercise [53–55].

One of the factors that could explain the lack of benefits observed with protein supplementation in the present study is the high daily protein intake of cyclists––*i.e*. >2.5 g/kg on average even in those in the placebo group, who were not taking the protein supplement––which was well above current recommendations for endurance athletes (1.2-1.4 g/kg) [56]. Previous studies have also reported very high protein intakes (from ~2.8 to ~3.8 g/kg per day) in professional cyclists during periods of high physical demands such as multistage races [39–42]. In this regard, meta-analytical evidence indicates that protein supplementation can promote training-induced adaptations (*e.g*. increases in muscle mass and strength) up to a total daily protein intake of 1.6 g/kg, beyond which no further benefits are observed [1]. Thus, although protein requirements in endurance athletes seem to be increased, particularly during periods of strenuous training loads such as those applied here [45,57], the amount of proteins present in the normal diet of our participants might have sufficed to meet physiological demands.

Some limitations of the present study should be acknowledged. We analyzed a reduced number of participants due to the inherent difficulties of conducting research with high-performance athletes such as those assessed here. Moreover, we used a parallel-group design, but a crossover design could have reduced the potential confounding effect of between-individual variability. Some of the outcome assessment methods used here could also be optimized. Notably, nutritional intake was registered with a self-reported food diary, and we did not use more accurate indicators of body compositions such as dual-energy X-ray absorptiometry. On the other hand, the high protein intake of the participants might have confounded our results, and it is possible to hypothesize that protein supplementation could have been beneficial if dietary protein intake had been restricted (*e.g*. <1.5 g/kg). However, the present study can be considered ecologically valid, as we did not modify participants’ habitual diet, which can be considered a strength. Moreover, the fact of having performed a nutritional intervention in highly trained cyclists and having conducted a three-arm randomized controlled trial under field (“real”) conditions accounting for the effect of protein timing can also be considered major strengths of the study.

## 5. Conclusions

Protein supplementation––40 g of casein before sleep or during the afternoon––seems to provide no beneficial effects compared to an isoenergetic carbohydrate placebo on makers of recovery, performance, or body composition during a six-day training camp in professional cyclists that were already consuming a high-protein diet (>2.5 g/kg per day). Further research is needed to confirm whether this strategy could have beneficial effects on endurance athletes with a lower dietary protein intake.
